# Syntheses and crystal structures of di­chlorido­bis(2,3-di­methyl­pyrazine-κ*N*)zinc(II) and *catena*-poly[[di­chlorido­zinc(II)]-μ-2,3-di­methyl­pyrazine-κ^2^*N*^1^:*N*^4^]

**DOI:** 10.1107/S205698902500619X

**Published:** 2025-07-17

**Authors:** Christian Näther, Gaurav Bhosekar

**Affiliations:** a Institut für Anorganische Chemie, Universität Kiel, Max-Eyth.-Str. 2, 24118 Kiel, Germany; b Suman Ramesh Tulsiani Technical Campus - Faculty of Engineering, Pune, India; University of Aberdeen, United Kingdom

**Keywords:** synthesis, crystal structure, coordination polymer, zinc chloride, 2,3-di­methyl­pyrazine

## Abstract

The reactions of zinc(II)chloride with 2,3-di­methyl­pyrazine (C_6_H_8_N_2_) in different ratios in aceto­nitrile lead to the formation of [ZnCl_2_(C_6_H_8_N_2_)_2_] (**1**) and [ZnCl_2_(C_6_H_8_N_2_)]_*n*_ (**2**). The asymmetric unit of **1** consists of one Zn cation located on a twofold rotation axis, one chloride anion and one 2,3-di­methyl­pyrazine ligand that occupy general positions. In compound **2** the asymmetric unit is built up of one zinc cation, two chloride anions and one 2,3-di­methyl­pyrazine ligand that are located in general positions in the uncommon trigonal space group *P*3_2_. In compound **1**, the Zn cations are tetra­hedrally coordinated by two chloride anions and two 2,3-di­methyl­pyrazine ligands forming discrete complexes. These complexes are arranged in columns that proceed along the *c*-axis direction. The Zn cations in **2** are also tetra­hedrally coordinated by two chloride anions and two 2,3-di­methyl­pyrazine ligands but linked *via* the bridging 2,3-di­methyl­pyrazine ligands into helical infinite chains that propagate along the *c*-axis direction. Powder X-ray diffraction measurements indicate that both compounds were obtained as pure crystalline phases.

## Abstract 2

The syntheses and crystal structures of two new ZnCl_2_–2,3-di­methyl­pyrazine (C_6_H_8_N_2_) coordination compounds with the composition ZnCl_2_(C_6_H_8_N_2_)_2_ (**1**) and ZnCl_2_((C_6_H_8_N_2_) (**2**) are reported. Compound **1** consists of discrete complexes, whereas in compound **2** the Zn cations are linked by the 2,3-di­methyl­pyrazine ligands into helical [001] chains.

## 1. Chemical context

Coordination compounds based on transition-metal halides and neutral coligands have been investigated for many years. Such compounds shows extremely versatile structural behavior, which is especially valid for compounds containing copper(I) cations (Kromp & Sheldrick, 1999 ▸; Peng *et al.*, 2010 ▸; Näther & Jess, 2002 ▸, 2004 ▸; Li *et al.*, 2005 ▸). In this class of compounds, the copper cations can be linked by the halide anions into dinuclear units, chains or layers, which can be additionally connected if bridging instead of mono-coordinating coligands are used. Moreover, for a specific copper(I) halide and a specific coligand, compounds with a different ratio between the copper(I) halide and the neutral coligand are observed. If larger amounts of the coligands are used in the synthesis, mostly discrete units are obtained and an excess of the copper(I) halide leads to the formation of more condensed networks. The latter compounds can also be obtained if the discrete compounds with larger amounts of the coligands are heated, which usually leads to a stepwise removal of the coligands and the formation of new compounds consisting of single and double chains or layers (Näther *et al.*, 2001 ▸, 2002 ▸).

In contrast, compounds based on divalent cations show a less pronounced structural variability. In most cases, the metal cations are linked by pairs of μ-1,1 bridging halide anions into chains and such chains can be further connected into layers if bridging coligands are used. This is the case, *e.g*. in Cd*X*_2_ compounds with the composition Cd*X*_2_(pyrazine) with *X* = Cl (Cambridge Structural Database refcode TISSUJ; Pickardt & Staub, 1996 ▸), *X* = Br (RINSIQ and RINSOW; Bailey & Pennington, 1997 ▸), and *X* = I (RINSIQ01 and RINSOW01; Pickardt & Staub, 1997 ▸), which have been known for many years. In these compounds, the Cd^2+^ cations are linked by pairs of halide anions into linear chains that are further connected into layers by the bridging pyrazine ligands.

In the course of our ongoing work in this area, we tried to prepare ZnCl_2_ compounds with 2,3-di­methyl­pyrazine (C_6_H_8_N_2_) that also can act as bridging ligands. This led to the formation of two different crystalline phases that were characterized by single crystal X-ray diffraction. Related compounds containing zinc and pyrazine are described in the *Database survey* section below.
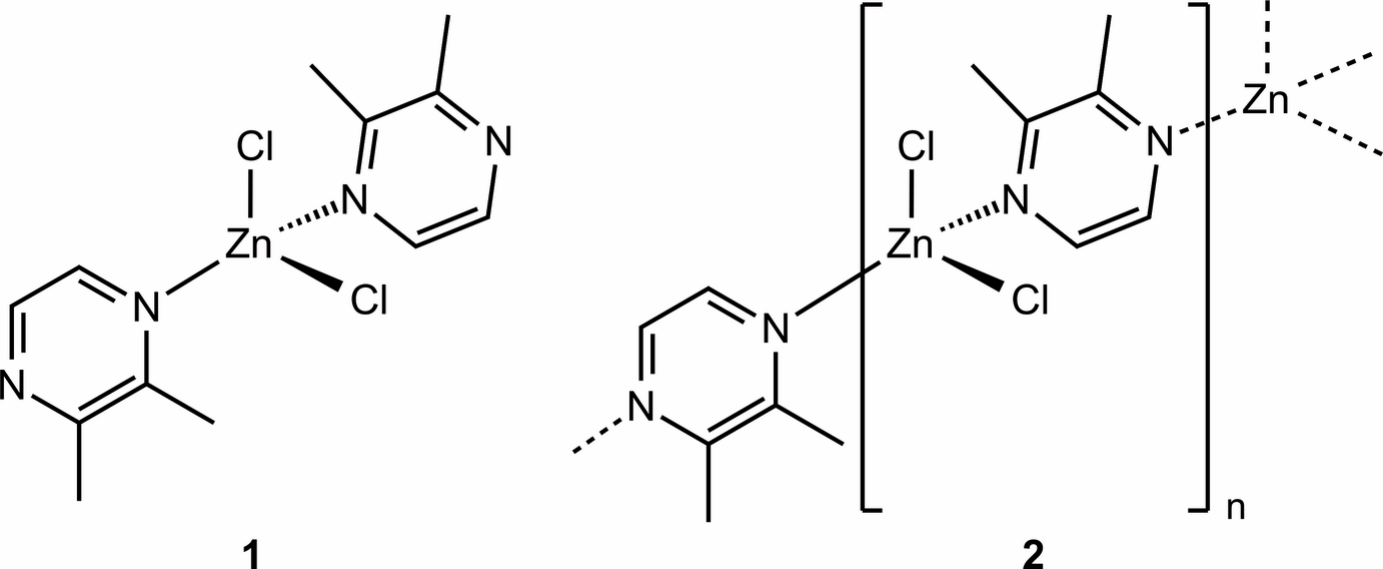


## 2. Structural commentary

**Figure 1:**
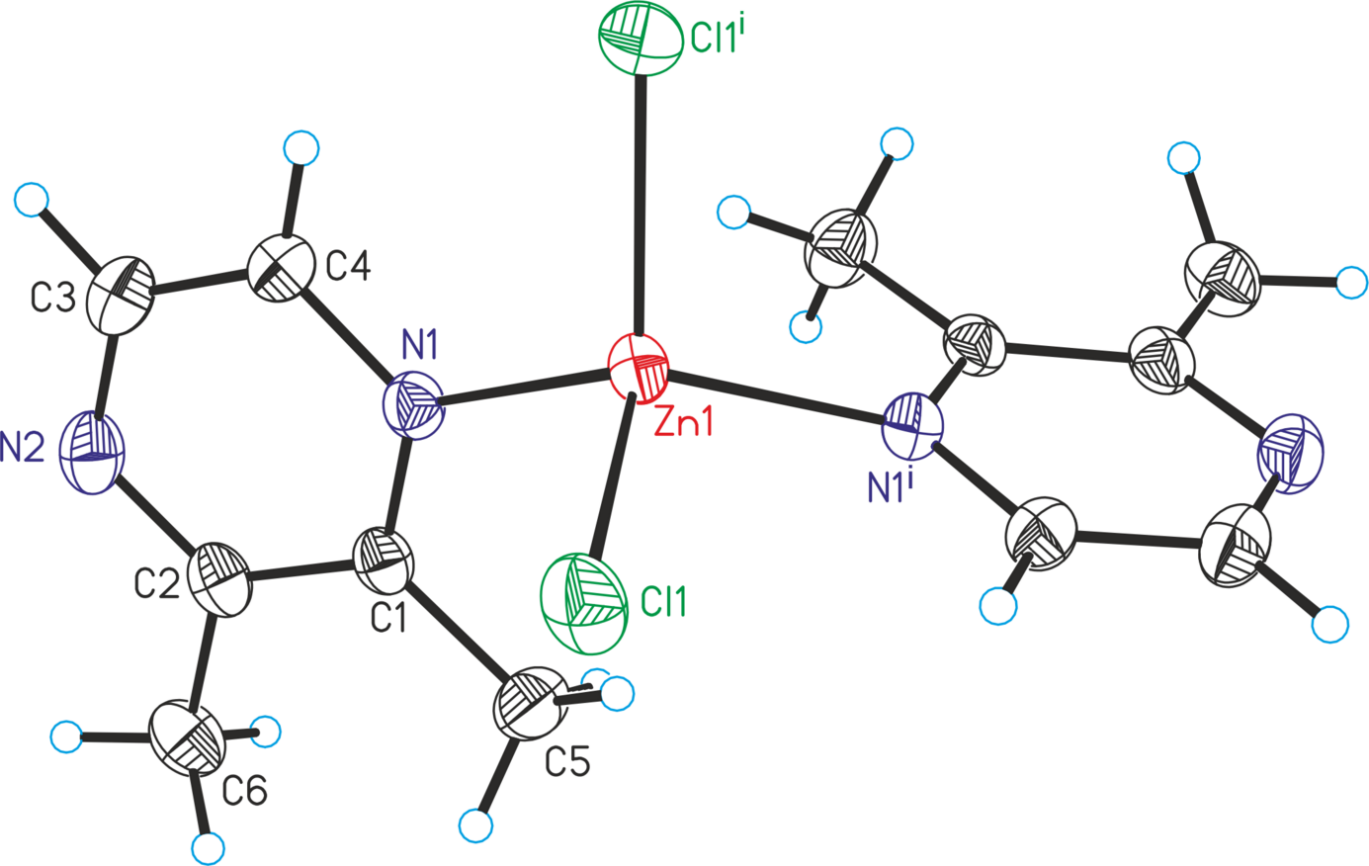
The mol­ecular structure of **1** with displacement ellipsoids drawn at the 50% probability level. Symmetry code for the generation of equivalent atoms: (i) −*x* + 1, *y*, −*z* + 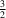.

The asymmetric unit of ZnCl_2_(C_6_H_8_N_2_)_2_ (**1**) consists of one Zn cation that is located on a twofold rotation axis, as well as one chloride anion and one 2,3-di­methyl­pyrazine ligand in general positions. The Zn cations are tetra­hedrally coordinated by two 2,3-di­methyl­pyrazine coligands and two chloride anions into discrete complexes (Fig. 1 ▸). The Cl—Zn—Cl and N—Zn—N angles are larger than the Cl—Zn—N angles, which shows that the tetra­hedra are slightly distorted (Table 1 ▸).

**Figure 2:**
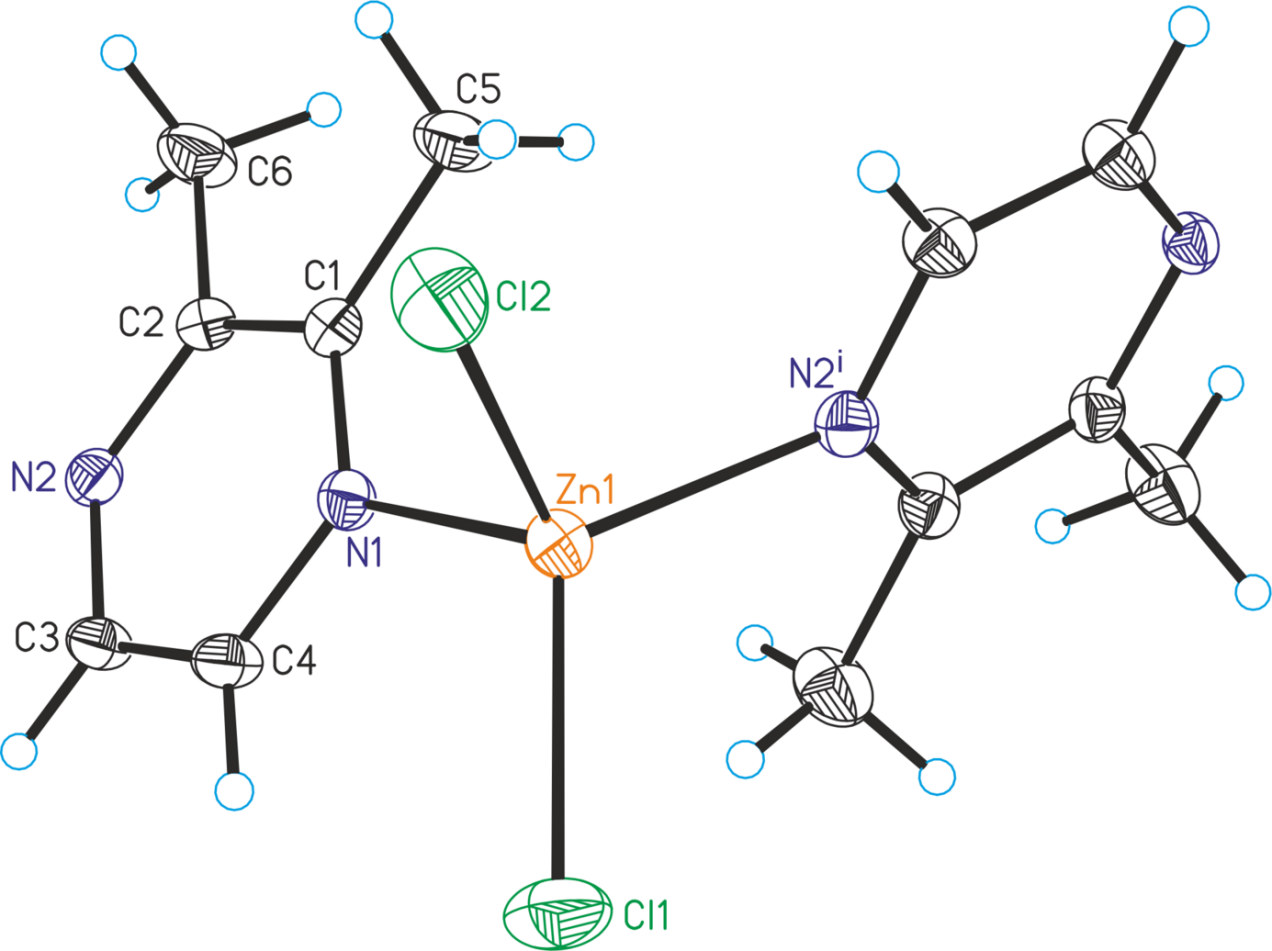
The mol­ecular structure of **2** with displacement ellipsoids drawn at the 50% probability level. Symmetry code for the generation of equivalent atoms: (i) −*x* + *y* + 1, −*x* + 1, *z* + 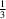.

**Figure 3:**
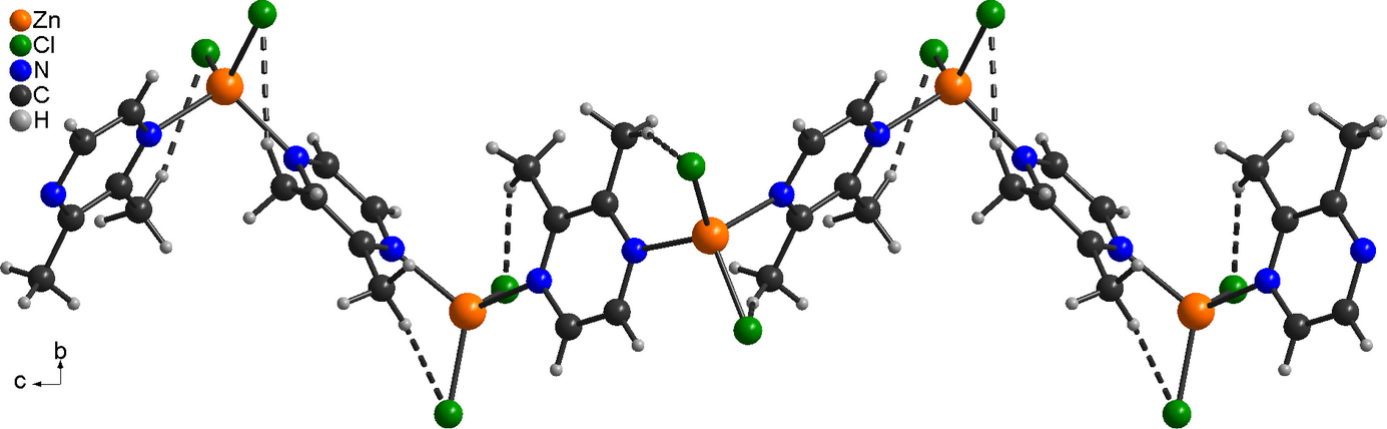
Part of a [001] chain in **2** with intra­chain and intra­chain C—H⋯Cl hydrogen bonds shown as dashed lines.

The asymmetric unit of [ZnCl_2_(C_6_H_8_N_2_)]_*n*_ (**2**) consists of one Zn cation, two chloride anions and one 2,3-di­methyl­pyrazine ligand, but in contrast to compound **1**, all atoms are located in general positions (Fig. 2 ▸). As in compound **1**, the Zn cations are terahedrally coordinated by two chloride anions and two 2,3-di­methyl­pyrazine ligands. In contrast to compound **1** the N—Zn—N angles are smaller than the N—Zn—Cl angles with the latter close to the ideal tetra­hedral values (Table 2 ▸). The Zn cations are linked into helical chains by the 2,3-di­methyl­pyrazine ligands and these chains propagate in the crystallographic *c*-axis direction (Fig. 3 ▸)

## 3. Supra­molecular features

**Figure 4:**
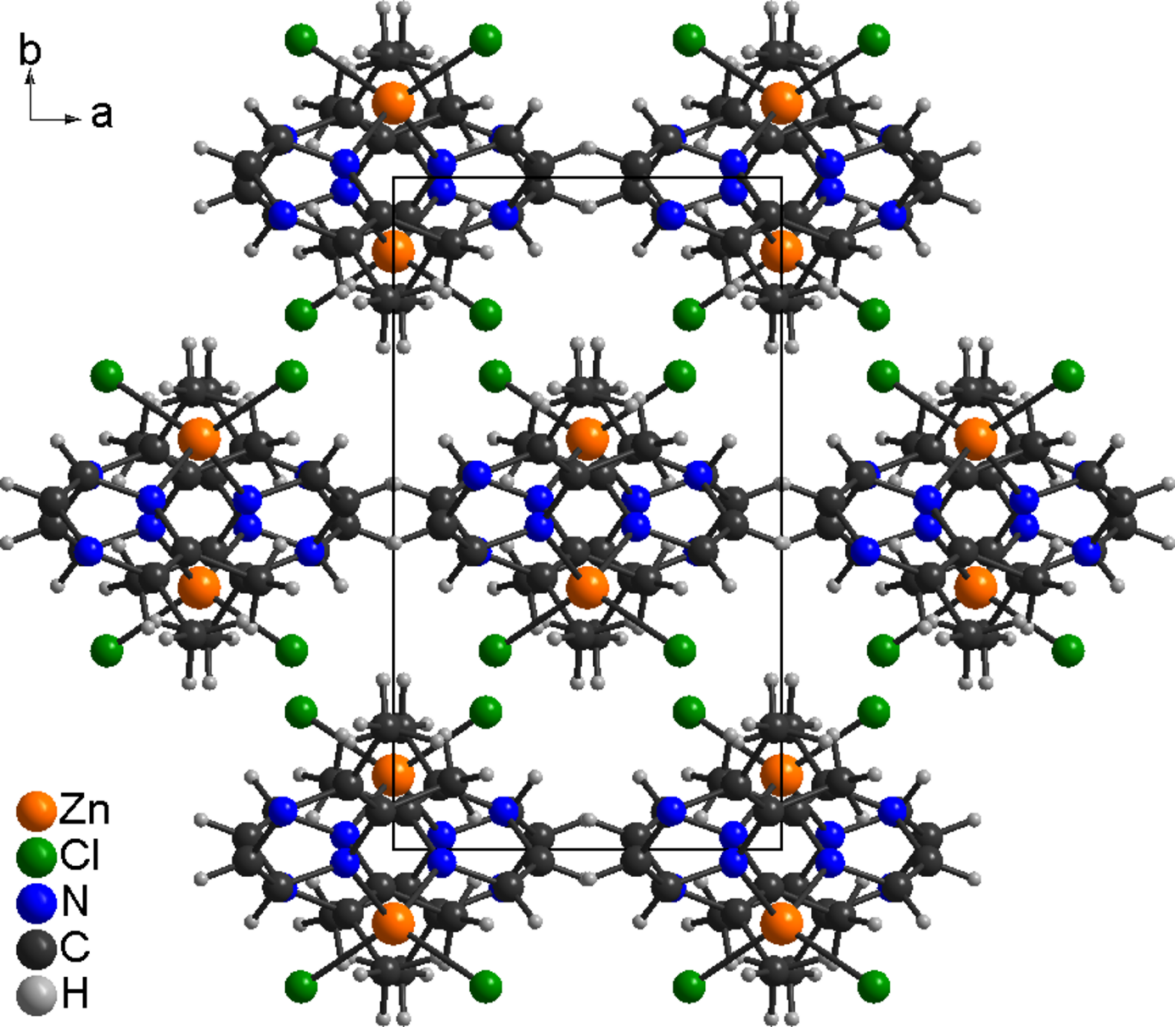
Crystal structure of **1** with view along the crystallographic *c*-axis direction.

In compound **1**, the discrete complexes are arranged into columns that propagate in the crystallographic *c*-axis direction (Fig. 4 ▸). Between these columns there are no pronounced inter­molecular inter­actions. One C—H⋯N and one C—H⋯Cl contact (Table 3 ▸) are observed, but at relatively long H⋯N and H⋯Cl distances and with angles far from linearity, which indicate that these are, at best, very weak inter­actions.

**Figure 5:**
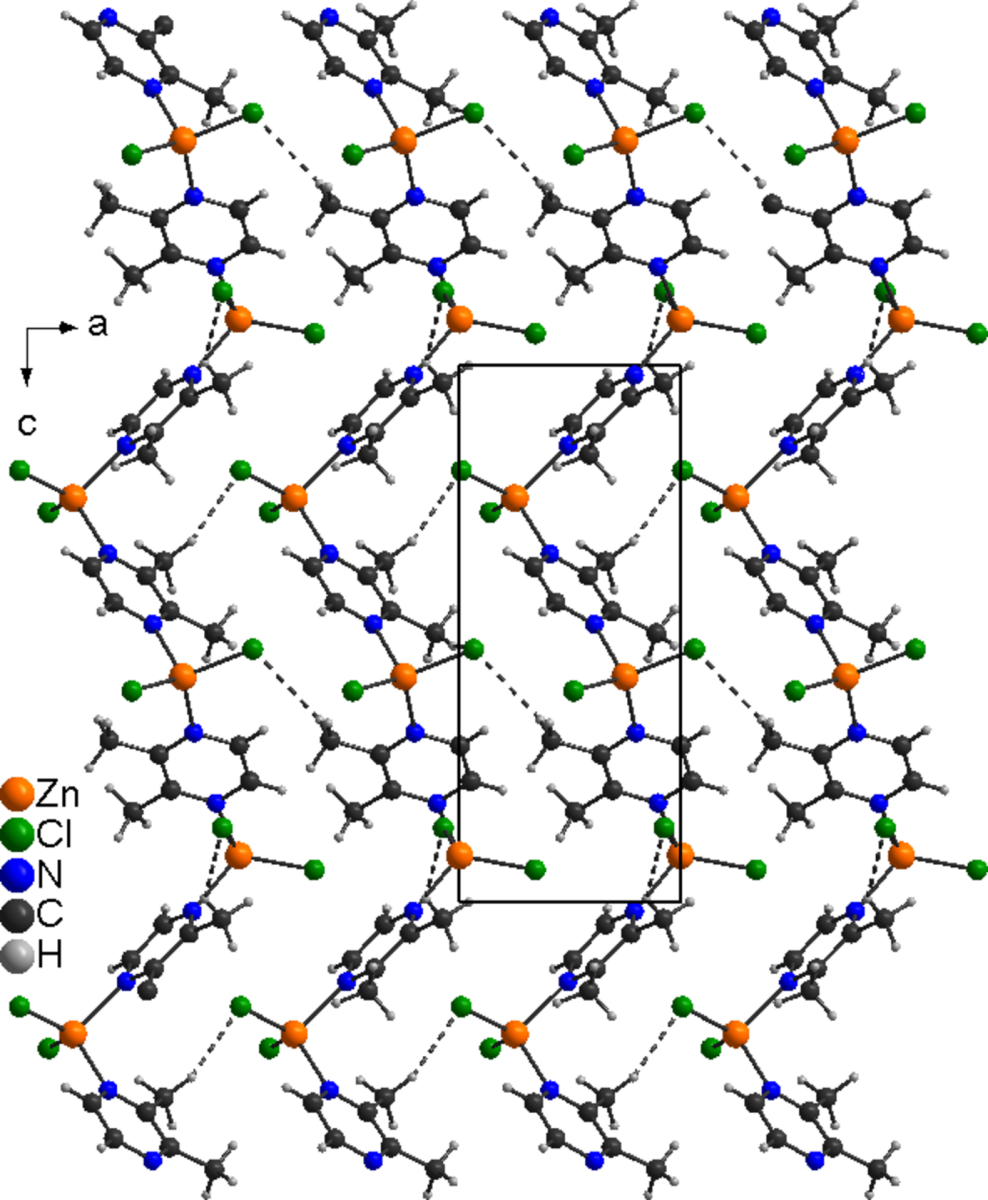
The crystal structure of **2** with view along the crystallographic *b*-axis direction and inter­chain C—H⋯Cl hydrogen bonding shown as dashed lines.

In contrast, in compound **2**, intra and inter­chain C—H⋯Cl hydrogen bonding is observed. Within the chains there are two C—H⋯Cl contacts between one H atom of the methyl groups and a halide anions but the C—H⋯Cl angles deviate from linearity, indicating that these are very weak inter­actions (Fig. 3 ▸ and Table 4 ▸). The chains are crosslinked by C—H⋯Cl contacts, but even here relatively long H⋯Cl distances and angles far from linearity are observed, indicating only weak inter­actions (Fig. 5 ▸ and Table 4 ▸).

## 4. Database survey

A search in the CCDC database (Groom *et al.*, 2016 ▸, CSD Version 5.43, January 2025) using CONQUEST (Bruno *et al.*, 2002 ▸) revealed that no compounds with twofold positively charged transition-metal halides and 2,3-di­methyl­pyrazine are known. However, with pyrazine (C_4_H_4_N_2_), Zn^2+^ cations and halide anions, a number of compounds with different stoichiometries and different structural behaviors are observed. With ZnCl_2_, two compounds with the composition ZnCl_2_(C_4_H_4_N_2_)_2_ (REMPAB; Bhosekar *et al.*, 2006 ▸) and ZnCl_2_(C_4_H_4_N_2_) (TISTAQ; Pickardt & Staub, 1996 ▸) have been reported. In the first compound, the Zn cations are octa­hedrally coordinated by two chloride anions and four pyrazine ligands and are linked into layers by the coligands. In the pyrazine-deficient compound, the Zn cations are also octa­hedrally coordinated but the Zn cations are linked by pairs of bridging halide anions into chains that are connected into layers by the coligands, as is the case in the corresponding Cd compounds. For ZnBr_2_(C_4_H_4_N_2_)_2_, two different modifications [EBOLAI (Bourne *et al.*, 2001 ▸) and EBOLAI01 (Bhosekar *et al.*, 2006 ▸)] are observed, of which one is isotypical to the corresponding chloride compounds. In both compounds, the same layer topology is observed. The crystal structure of ZnBr_2_(C_4_H_4_N_2_) is different from that of the chloride compounds. In this compound, the Zn cations are tetra­hedrally coordinated and linked into corrugated chains *via* the neutral coligands (EBOKUB; Bourne *et al.*, 2001 ▸). Finally, ZnI_2_(C_4_H_4_N_2_) is also known and shows a structure similar to that of the corresponding bromide compound with a tetra­hedral coordination of the metal center [ISOPOV (Song *et al.*, 2004 ▸) and ISOPOV01 (Bhosekar *et al.*, 2006 ▸)].

## 5. Synthesis and crystallization

Zinc chloride and 2,3-di­methyl­pyrazine were purchased from Sigma-Aldrich. To prepare **1**, 1.00 mmol (136.3 mg) of zinc chloride was reacted with 2.00 mmol (216.3 mg) of 2,3-di­methyl­pyrazine in 1 ml of aceto­nitrile. The reaction mixture was stirred for 2 d and the precipitate was filtered off and dried. Single crystals were obtained under the same reaction conditions without stirring. Compound **2** was prepared when 1.00 mmol (136.3 mg) of zinc chloride was reacted with 1.00 mmol (108.1 mg) of 2,3-di­methyl­pyrazine in 1 ml of aceto­nitrile. The reaction mixture was stirred for 2 d and the precipitate was filtered off and dried. Single crystals were obtained under the same reaction conditions without stirring.

**Figure 6:**
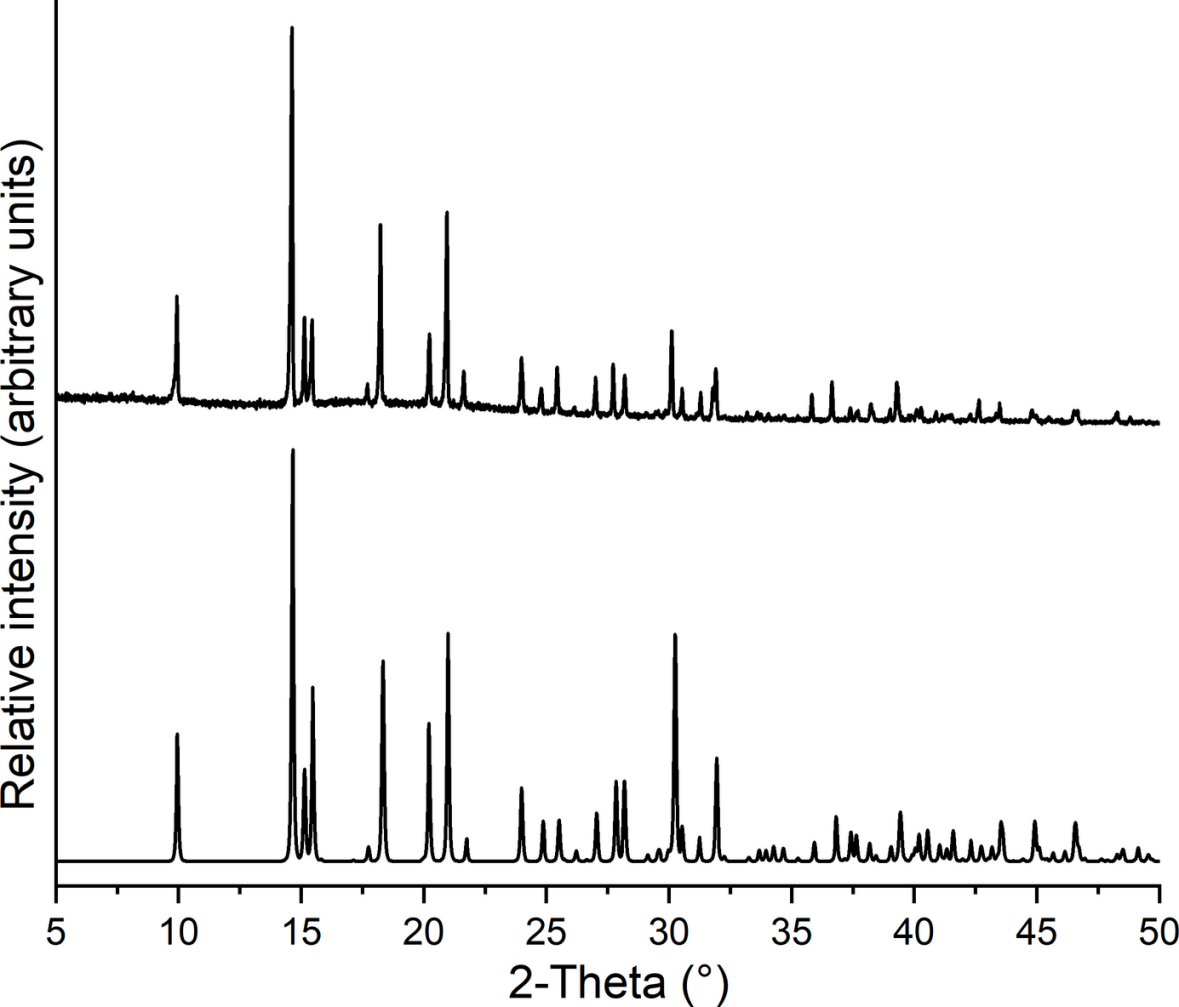
Experimental (top) and calculated X-ray powder pattern (bottom) of **1**.

Comparison of the the experimental X-ray powder patterns with that calculated for the title compounds from single-crystal data shows that pure crystalline phases have been obtained (Figs. 6 ▸ and 7 ▸). The PXRD measurements were performed with Cu *K*α_1_ radiation (λ = 1.540598 Å) using a Stoe Transmission Powder Diffraction System (STADI P) equipped with a MYTHEN 1K detector and a Johansson-type Ge(111) monochromator.

## 6. Refinement

Crystal data, data collection and structure refinement details are summarized in Table 5 ▸. The C—H hydrogen atoms were positioned with idealized geometry (methyl H atoms allowed to rotate but not to tip) and were refined isotropically with *U*_iso_(H) = 1.2 *U*_eq_(C) (1.5 for methyl H atoms).

## Supplementary Material

Crystal structure: contains datablock(s) 1, 2, global. DOI: 10.1107/S205698902500619X/hb8145sup1.cif

Structure factors: contains datablock(s) 1. DOI: 10.1107/S205698902500619X/hb81451sup2.hkl

Structure factors: contains datablock(s) 2. DOI: 10.1107/S205698902500619X/hb81452sup3.hkl

CCDC references: 2472527, 2472528

Additional supporting information:  crystallographic information; 3D view; checkCIF report

## Figures and Tables

**Figure 7:**
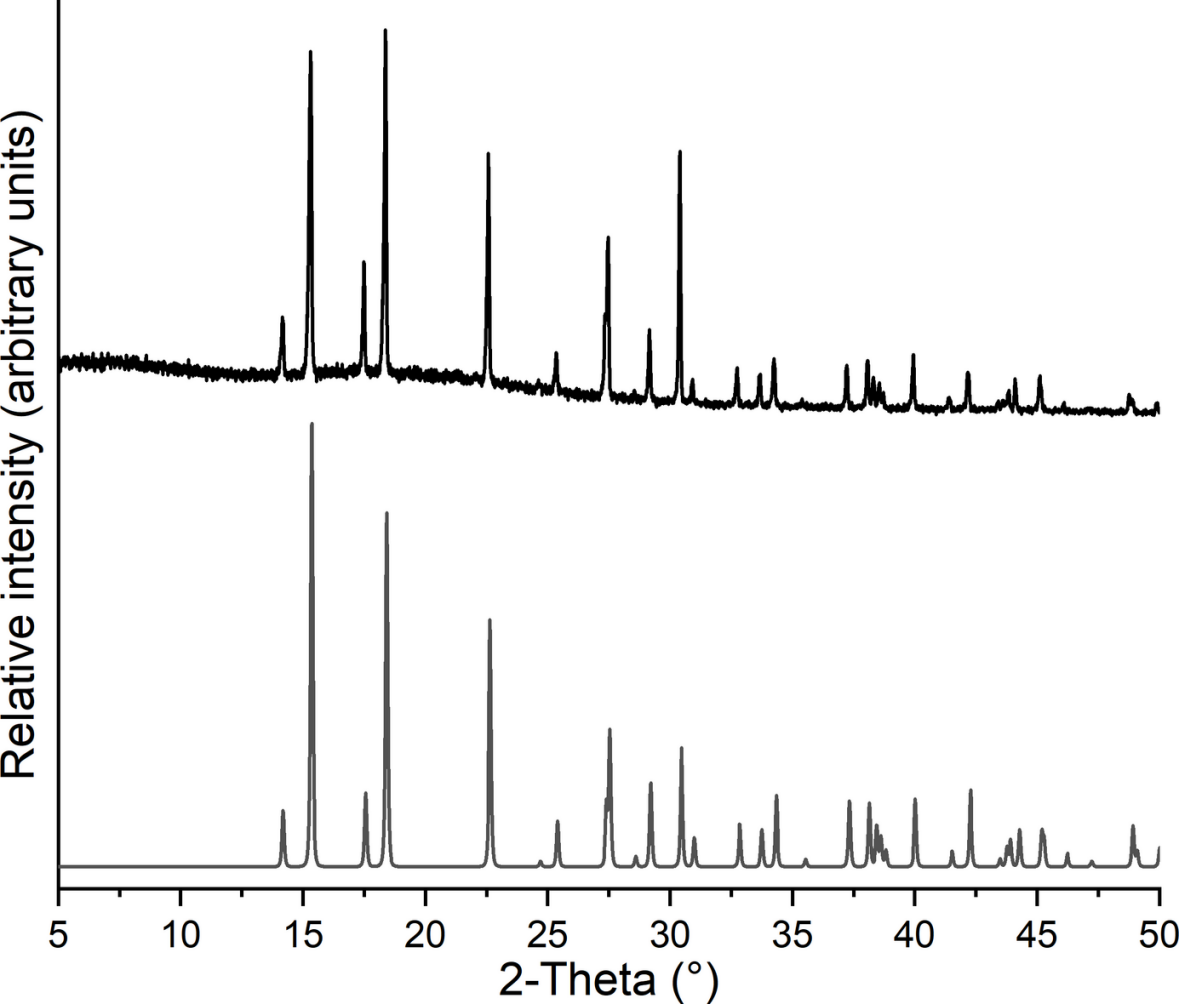
Experimental (top) and calculated X-ray powder pattern (bottom) of **2**.

**Table 1:** Selected geometric parameters (Å, °) for **1**[Chem scheme1]

Zn1—Cl1	2.2261 (7)	Zn1—N1	2.0769 (19)
			
Cl1^i^—Zn1—Cl1	118.70 (4)	N1—Zn1—Cl1^i^	105.92 (6)
N1—Zn1—Cl1	105.21 (6)	N1^i^—Zn1—N1	116.47 (11)

Symmetry code: (i) 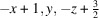.

**Table 2:** Selected geometric parameters (Å, °) for **2**[Chem scheme1]

Zn1—Cl1	2.2142 (11)	Zn1—N1	2.109 (3)
Zn1—Cl2	2.2042 (11)	Zn1—N2^i^	2.083 (3)
			
Cl2—Zn1—Cl1	116.23 (5)	N2^i^—Zn1—Cl1	115.43 (10)
N1—Zn1—Cl1	107.11 (9)	N2^i^—Zn1—Cl2	107.98 (10)
N1—Zn1—Cl2	105.99 (9)	N2^i^—Zn1—N1	102.82 (12)

Symmetry code: (i) 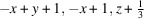.

**Table 3:** Hydrogen-bond geometry (Å, °) for **1**[Chem scheme1]

*D*—H⋯*A*	*D*—H	H⋯*A*	*D*⋯*A*	*D*—H⋯*A*
C3—H3⋯N2^ii^	0.94	2.68	3.459 (3)	140
C4—H4⋯Cl1^i^	0.94	2.81	3.445 (3)	126

Symmetry codes: (i) 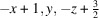; (ii) 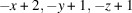.

**Table 4:** Hydrogen-bond geometry (Å, °) for **2**[Chem scheme1]

*D*—H⋯*A*	*D*—H	H⋯*A*	*D*⋯*A*	*D*—H⋯*A*
C3—H3⋯Cl1^ii^	0.94	2.90	3.590 (4)	132
C3—H3⋯Cl2^iii^	0.94	2.85	3.482 (4)	126
C4—H4⋯Cl1	0.94	2.87	3.500 (4)	126
C5—H5*A*⋯Cl2	0.97	2.85	3.724 (5)	151
C6—H6*A*⋯Cl1^iii^	0.97	2.80	3.721 (5)	160
C6—H6*C*⋯Cl2^iv^	0.97	2.80	3.596 (5)	140

Symmetry codes: (ii) 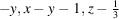; (iii) 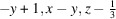; (iv) 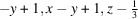.

**Table 5:** Experimental details

	**1**	**2**
Crystal data		
Chemical formula	[ZnCl_2_(C_6_H_8_N_2_)_2_]	[ZnCl_2_(C_6_H_8_N_2_)]
*M*_r_	352.56	244.41
Crystal system, space group	Monoclinic, *C*2/*c*	Trigonal, *P*3_2_
Temperature (K)	220	220
*a*, *b*, *c* (Å)	6.9984 (4), 12.0864 (9), 17.8220 (12)	7.2027 (5), 7.2027 (5), 15.1418 (12)
α, β, γ (°)	90, 94.773 (8), 90	90, 90, 120
*V* (Å^3^)	1502.25 (17)	680.30 (11)
*Z*	4	3
Radiation type	Mo *K*α	Mo *K*α
μ (mm^−1^)	1.98	3.23
Crystal size (mm)	0.11 × 0.08 × 0.06	0.12 × 0.07 × 0.05

Data collection		
Diffractometer	Stoe *IPDS2*	Stoe *IPDS2*
Absorption correction	Numerical (*X-RED* and *X-SHAPE*; Stoe, 2008 ▸)	Numerical (*X-RED* and *X-SHAPE*; Stoe, 2008 ▸)
*T*_min_, *T*_max_	0.684, 0.802	0.530, 0.709
No. of measured, independent and observed [*I* > 2σ(*I*)] reflections	6648, 1822, 1593	4933, 2178, 2078
*R*_int_	0.031	0.037
(sin θ/λ)_max_ (Å^−1^)	0.663	0.660

Refinement		
*R*[*F*^2^ > 2σ(*F*^2^)], *wR*(*F*^2^), *S*	0.035, 0.093, 1.06	0.026, 0.066, 1.02
No. of reflections	1822	2178
No. of parameters	90	103
No. of restraints	0	1
H-atom treatment	H-atom parameters constrained	H-atom parameters constrained
Δρ_max_, Δρ_min_ (e Å^−3^)	0.41, −0.47	0.43, −0.45
Absolute structure	–	Flack *x* determined using 989 quotients [(*I*^+^)−(*I*^−^)]/[(*I*^+^)+(*I*^−^)] (Parsons *et al.*, 2013 ▸)
Absolute structure parameter	–	−0.008 (9)

Computer programs: *X-AREA* (Stoe, 2008 ▸), *SHELXT2014/4* (Sheldrick, 2015*a* ▸), *SHELXL2016/6* (Sheldrick, 2015*b* ▸), *DIAMOND* (Brandenburg, 1999 ▸) and *XP* in *SHELXTL-PC* (Sheldrick, 2008 ▸), *publCIF* (Westrip, 2010 ▸).

## supplementary crystallographic information

### Dichloridobis(2,3-dimethylpyrazine-κN)zinc(II) (1) Crystal data

**Table:**

[ZnCl_2_(C_6_H_8_N_2_)_2_]	*F*(000) = 720
*M**_r_* = 352.56	*D*_x_ = 1.559 Mg m^−^^3^
Monoclinic, *C*2/*c*	Mo *K*α radiation, λ = 0.71073 Å
*a* = 6.9984 (4) Å	Cell parameters from 6648 reflections
*b* = 12.0864 (9) Å	θ = 2.3–28.1°
*c* = 17.8220 (12) Å	µ = 1.98 mm^−^^1^
β = 94.773 (8)°	*T* = 220 K
*V* = 1502.25 (17) Å^3^	Block, colorless
*Z* = 4	0.11 × 0.08 × 0.06 mm

### Dichloridobis(2,3-dimethylpyrazine-κN)zinc(II) (1) Data collection

**Table:**

Stoe IPDS-2 diffractometer	1593 reflections with *I* > 2σ(*I*)
Graphite monochromator	*R*_int_ = 0.031
ω scans	θ_max_ = 28.1°, θ_min_ = 2.3°
Absorption correction: numerical (X-Red and X-Shape; Stoe, 2008)	*h* = −8→9
*T*_min_ = 0.684, *T*_max_ = 0.802	*k* = −15→15
6648 measured reflections	*l* = −23→23
1822 independent reflections	

### Dichloridobis(2,3-dimethylpyrazine-κN)zinc(II) (1) Refinement

**Table:**

Refinement on *F*^2^	Hydrogen site location: inferred from neighbouring sites
Least-squares matrix: full	H-atom parameters constrained
*R*[*F*^2^ > 2σ(*F*^2^)] = 0.035	*w* = 1/[σ^2^(*F*_o_^2^) + (0.0513*P*)^2^ + 2.9305*P*] where *P* = (*F*_o_^2^ + 2*F*_c_^2^)/3
*wR*(*F*^2^) = 0.093	(Δ/σ)_max_ < 0.001
*S* = 1.06	Δρ_max_ = 0.41 e Å^−^^3^
1822 reflections	Δρ_min_ = −0.47 e Å^−^^3^
90 parameters	Extinction correction: [(*I*^+^)-(*I*^-^)]/[(*I*^+^)+(*I*^-^)], Fc^*^=kFc[1+0.001xFc^2^λ^3^/sin(2θ)]^-1/4^
0 restraints	Extinction coefficient: 0.0093 (10)
Primary atom site location: dual	

### Dichloridobis(2,3-dimethylpyrazine-κN)zinc(II) (1) Special details

**Table:**

Geometry. All esds (except the esd in the dihedral angle between two l.s. planes) are estimated using the full covariance matrix. The cell esds are taken into account individually in the estimation of esds in distances, angles and torsion angles; correlations between esds in cell parameters are only used when they are defined by crystal symmetry. An approximate (isotropic) treatment of cell esds is used for estimating esds involving l.s. planes.

### Dichloridobis(2,3-dimethylpyrazine-κN)zinc(II) (1) Fractional atomic coordinates and isotropic or equivalent isotropic displacement parameters (Å^2^)

**Table:**

	*x*	*y*	*z*	*U*_iso_*/*U*_eq_	
Zn1	0.500000	0.61053 (3)	0.750000	0.02169 (16)	
Cl1	0.26198 (10)	0.70442 (6)	0.68863 (4)	0.0358 (2)	
N1	0.6245 (3)	0.52007 (17)	0.66778 (11)	0.0223 (4)	
N2	0.7842 (3)	0.44320 (18)	0.54064 (12)	0.0296 (5)	
C1	0.5340 (3)	0.44317 (19)	0.62405 (13)	0.0218 (5)	
C2	0.6168 (4)	0.40470 (19)	0.55934 (14)	0.0252 (5)	
C3	0.8750 (4)	0.5175 (2)	0.58671 (15)	0.0300 (5)	
H3	0.995471	0.544204	0.575367	0.036*	
C4	0.7971 (3)	0.5558 (2)	0.64998 (14)	0.0266 (5)	
H4	0.865143	0.607572	0.681208	0.032*	
C5	0.3471 (4)	0.3998 (2)	0.64656 (16)	0.0322 (6)	
H5A	0.368112	0.329850	0.672689	0.048*	
H5B	0.259902	0.388697	0.601969	0.048*	
H5C	0.291943	0.452534	0.679620	0.048*	
C6	0.5205 (4)	0.3189 (2)	0.50888 (16)	0.0330 (6)	
H6A	0.585136	0.313807	0.462928	0.050*	
H6B	0.387536	0.339443	0.496697	0.050*	
H6C	0.526353	0.247889	0.534301	0.050*	

### Dichloridobis(2,3-dimethylpyrazine-κN)zinc(II) (1) Atomic displacement parameters (Å^2^)

**Table:**

	*U*^11^	*U*^22^	*U*^33^	*U*^12^	*U*^13^	*U*^23^
Zn1	0.0234 (2)	0.0244 (2)	0.0175 (2)	0.000	0.00345 (13)	0.000
Cl1	0.0373 (4)	0.0406 (4)	0.0294 (3)	0.0133 (3)	0.0023 (3)	0.0082 (3)
N1	0.0223 (9)	0.0249 (9)	0.0201 (9)	0.0021 (7)	0.0036 (7)	−0.0007 (8)
N2	0.0328 (11)	0.0299 (11)	0.0274 (11)	0.0058 (9)	0.0092 (8)	0.0017 (9)
C1	0.0250 (11)	0.0211 (10)	0.0192 (10)	0.0030 (8)	0.0011 (8)	0.0032 (9)
C2	0.0318 (12)	0.0218 (11)	0.0219 (11)	0.0057 (9)	0.0020 (9)	0.0023 (9)
C3	0.0270 (12)	0.0314 (13)	0.0327 (13)	−0.0003 (10)	0.0094 (10)	0.0015 (11)
C4	0.0218 (11)	0.0318 (12)	0.0265 (12)	−0.0021 (9)	0.0035 (9)	0.0002 (10)
C5	0.0275 (12)	0.0339 (14)	0.0356 (14)	−0.0042 (10)	0.0061 (10)	−0.0043 (11)
C6	0.0416 (15)	0.0287 (12)	0.0282 (13)	0.0029 (11)	−0.0008 (11)	−0.0033 (10)

### Dichloridobis(2,3-dimethylpyrazine-κN)zinc(II) (1) Geometric parameters (Å, º)

**Table:**

Zn1—Cl1^i^	2.2261 (7)	C2—C6	1.496 (4)
Zn1—Cl1	2.2261 (7)	C3—H3	0.9400
Zn1—N1^i^	2.077 (2)	C3—C4	1.373 (4)
Zn1—N1	2.0769 (19)	C4—H4	0.9400
N1—C1	1.338 (3)	C5—H5A	0.9700
N1—C4	1.346 (3)	C5—H5B	0.9700
N2—C2	1.329 (3)	C5—H5C	0.9700
N2—C3	1.341 (4)	C6—H6A	0.9700
C1—C2	1.412 (3)	C6—H6B	0.9700
C1—C5	1.495 (3)	C6—H6C	0.9700
			
Cl1^i^—Zn1—Cl1	118.70 (4)	N2—C3—C4	121.9 (2)
N1—Zn1—Cl1	105.21 (6)	C4—C3—H3	119.1
N1^i^—Zn1—Cl1^i^	105.21 (6)	N1—C4—C3	120.7 (2)
N1—Zn1—Cl1^i^	105.92 (6)	N1—C4—H4	119.6
N1^i^—Zn1—Cl1	105.92 (6)	C3—C4—H4	119.6
N1^i^—Zn1—N1	116.47 (11)	C1—C5—H5A	109.5
C1—N1—Zn1	124.81 (16)	C1—C5—H5B	109.5
C1—N1—C4	118.4 (2)	C1—C5—H5C	109.5
C4—N1—Zn1	115.78 (17)	H5A—C5—H5B	109.5
C2—N2—C3	117.6 (2)	H5A—C5—H5C	109.5
N1—C1—C2	119.9 (2)	H5B—C5—H5C	109.5
N1—C1—C5	118.0 (2)	C2—C6—H6A	109.5
C2—C1—C5	122.1 (2)	C2—C6—H6B	109.5
N2—C2—C1	121.3 (2)	C2—C6—H6C	109.5
N2—C2—C6	117.0 (2)	H6A—C6—H6B	109.5
C1—C2—C6	121.6 (2)	H6A—C6—H6C	109.5
N2—C3—H3	119.1	H6B—C6—H6C	109.5
			
Zn1—N1—C1—C2	165.76 (16)	C2—N2—C3—C4	−1.9 (4)
Zn1—N1—C1—C5	−14.9 (3)	C3—N2—C2—C1	2.0 (4)
Zn1—N1—C4—C3	−166.6 (2)	C3—N2—C2—C6	−177.9 (2)
N1—C1—C2—N2	0.1 (3)	C4—N1—C1—C2	−2.4 (3)
N1—C1—C2—C6	−179.9 (2)	C4—N1—C1—C5	176.9 (2)
N2—C3—C4—N1	−0.4 (4)	C5—C1—C2—N2	−179.2 (2)
C1—N1—C4—C3	2.6 (4)	C5—C1—C2—C6	0.8 (4)

Symmetry code: (i) −*x*+1, *y*, −*z*+3/2.

### Dichloridobis(2,3-dimethylpyrazine-κN)zinc(II) (1) Hydrogen-bond geometry (Å, º)

**Table:**

*D*—H···*A*	*D*—H	H···*A*	*D*···*A*	*D*—H···*A*
C3—H3···N2^ii^	0.94	2.68	3.459 (3)	140
C4—H4···Cl1^i^	0.94	2.81	3.445 (3)	126

Symmetry codes: (i) −*x*+1, *y*, −*z*+3/2; (ii) −*x*+2, −*y*+1, −*z*+1.

### catena-Poly[[dichloridozinc(II)]-µ-2,3-dimethylpyrazine-κ2N1:N4] (2) Crystal data

**Table:**

[ZnCl_2_(C_6_H_8_N_2_)]	*D*_x_ = 1.790 Mg m^−^^3^
*M**_r_* = 244.41	Mo *K*α radiation, λ = 0.71073 Å
Trigonal, *P*3_2_	Cell parameters from 4933 reflections
*a* = 7.2027 (5) Å	θ = 3.3–28.0°
*c* = 15.1418 (12) Å	µ = 3.23 mm^−^^1^
*V* = 680.30 (11) Å^3^	*T* = 220 K
*Z* = 3	Block, colorless
*F*(000) = 366	0.12 × 0.07 × 0.05 mm

### catena-Poly[[dichloridozinc(II)]-µ-2,3-dimethylpyrazine-κ2N1:N4] (2) Data collection

**Table:**

Stoe IPDS-2 diffractometer	2078 reflections with *I* > 2σ(*I*)
ω scans	*R*_int_ = 0.037
Absorption correction: numerical (X-Red and X-Shape; Stoe, 2008)	θ_max_ = 28.0°, θ_min_ = 3.3°
*T*_min_ = 0.530, *T*_max_ = 0.709	*h* = −9→9
4933 measured reflections	*k* = −9→9
2178 independent reflections	*l* = −19→19

### catena-Poly[[dichloridozinc(II)]-µ-2,3-dimethylpyrazine-κ2N1:N4] (2) Refinement

**Table:**

Refinement on *F*^2^	H-atom parameters constrained
Least-squares matrix: full	*w* = 1/[σ^2^(*F*_o_^2^) + (0.0475*P*)^2^] where *P* = (*F*_o_^2^ + 2*F*_c_^2^)/3
*R*[*F*^2^ > 2σ(*F*^2^)] = 0.026	(Δ/σ)_max_ < 0.001
*wR*(*F*^2^) = 0.066	Δρ_max_ = 0.43 e Å^−^^3^
*S* = 1.02	Δρ_min_ = −0.45 e Å^−^^3^
2178 reflections	Extinction correction: SHELXL-2016/6 (Sheldrick 2016), Fc^*^=kFc[1+0.001xFc^2^λ^3^/sin(2θ)]^-1/4^
103 parameters	Extinction coefficient: 0.059 (5)
1 restraint	Absolute structure: Flack *x* determined using 989 quotients [(*I*^+^)-(*I*^-^)]/[(*I*^+^)+(*I*^-^)] (Parsons *et al.*, 2013)
Primary atom site location: dual	Absolute structure parameter: −0.008 (9)
Hydrogen site location: inferred from neighbouring sites	

### catena-Poly[[dichloridozinc(II)]-µ-2,3-dimethylpyrazine-κ2N1:N4] (2) Special details

**Table:**

Geometry. All esds (except the esd in the dihedral angle between two l.s. planes) are estimated using the full covariance matrix. The cell esds are taken into account individually in the estimation of esds in distances, angles and torsion angles; correlations between esds in cell parameters are only used when they are defined by crystal symmetry. An approximate (isotropic) treatment of cell esds is used for estimating esds involving l.s. planes.

### catena-Poly[[dichloridozinc(II)]-µ-2,3-dimethylpyrazine-κ2N1:N4] (2) Fractional atomic coordinates and isotropic or equivalent isotropic displacement parameters (Å^2^)

**Table:**

	*x*	*y*	*z*	*U*_iso_*/*U*_eq_	
Zn1	0.25122 (6)	−0.00067 (6)	0.24667 (2)	0.01715 (15)	
Cl1	0.13944 (19)	−0.34299 (16)	0.27312 (8)	0.0330 (3)	
Cl2	0.00920 (17)	0.07332 (19)	0.19522 (8)	0.0310 (3)	
N1	0.4909 (5)	0.1016 (5)	0.1488 (2)	0.0154 (6)	
N2	0.7919 (5)	0.2020 (5)	0.0175 (2)	0.0169 (6)	
C1	0.6175 (6)	0.3059 (6)	0.1242 (3)	0.0170 (7)	
C2	0.7759 (6)	0.3587 (6)	0.0583 (3)	0.0171 (7)	
C3	0.6607 (6)	−0.0026 (6)	0.0417 (3)	0.0179 (7)	
H3	0.669885	−0.113306	0.012859	0.022*	
C4	0.5131 (6)	−0.0516 (6)	0.1080 (3)	0.0184 (7)	
H4	0.425794	−0.195318	0.125101	0.022*	
C5	0.5867 (7)	0.4770 (6)	0.1651 (3)	0.0270 (9)	
H5A	0.442266	0.414171	0.188400	0.041*	
H5B	0.608877	0.583536	0.120896	0.041*	
H5C	0.689115	0.544493	0.212727	0.041*	
C6	0.9230 (8)	0.5846 (7)	0.0315 (3)	0.0288 (9)	
H6A	1.043882	0.593490	−0.000465	0.043*	
H6B	0.974247	0.674413	0.083733	0.043*	
H6C	0.846871	0.633232	−0.006131	0.043*	

### catena-Poly[[dichloridozinc(II)]-µ-2,3-dimethylpyrazine-κ2N1:N4] (2) Atomic displacement parameters (Å^2^)

**Table:**

	*U*^11^	*U*^22^	*U*^33^	*U*^12^	*U*^13^	*U*^23^
Zn1	0.0168 (2)	0.0170 (2)	0.0174 (2)	0.00821 (17)	−0.00047 (15)	−0.00136 (15)
Cl1	0.0359 (6)	0.0185 (4)	0.0402 (7)	0.0104 (4)	0.0011 (4)	0.0064 (4)
Cl2	0.0266 (5)	0.0401 (6)	0.0340 (6)	0.0224 (4)	−0.0108 (4)	−0.0091 (4)
N1	0.0164 (14)	0.0157 (14)	0.0139 (15)	0.0080 (12)	0.0024 (11)	0.0004 (11)
N2	0.0176 (14)	0.0171 (15)	0.0160 (15)	0.0086 (12)	−0.0011 (12)	0.0010 (12)
C1	0.0205 (17)	0.0168 (16)	0.0143 (16)	0.0099 (14)	0.0002 (13)	0.0012 (13)
C2	0.0208 (17)	0.0152 (16)	0.0165 (17)	0.0100 (14)	−0.0001 (14)	0.0013 (13)
C3	0.0204 (17)	0.0139 (16)	0.0208 (18)	0.0096 (14)	0.0011 (14)	−0.0004 (13)
C4	0.0209 (18)	0.0144 (16)	0.0217 (19)	0.0101 (14)	0.0031 (15)	0.0018 (13)
C5	0.037 (2)	0.0172 (18)	0.029 (2)	0.0152 (17)	0.0098 (18)	0.0027 (16)
C6	0.035 (2)	0.0155 (17)	0.031 (2)	0.0097 (17)	0.0089 (18)	0.0008 (16)

### catena-Poly[[dichloridozinc(II)]-µ-2,3-dimethylpyrazine-κ2N1:N4] (2) Geometric parameters (Å, º)

**Table:**

Zn1—Cl1	2.2142 (11)	C2—C6	1.487 (5)
Zn1—Cl2	2.2042 (11)	C3—H3	0.9400
Zn1—N1	2.109 (3)	C3—C4	1.374 (6)
Zn1—N2^i^	2.083 (3)	C4—H4	0.9400
N1—C1	1.339 (5)	C5—H5A	0.9700
N1—C4	1.341 (5)	C5—H5B	0.9700
N2—C2	1.340 (5)	C5—H5C	0.9700
N2—C3	1.344 (5)	C6—H6A	0.9700
C1—C2	1.418 (5)	C6—H6B	0.9700
C1—C5	1.491 (5)	C6—H6C	0.9700
			
Cl2—Zn1—Cl1	116.23 (5)	N2—C3—H3	119.7
N1—Zn1—Cl1	107.11 (9)	N2—C3—C4	120.7 (4)
N1—Zn1—Cl2	105.99 (9)	C4—C3—H3	119.7
N2^i^—Zn1—Cl1	115.43 (10)	N1—C4—C3	121.3 (4)
N2^i^—Zn1—Cl2	107.98 (10)	N1—C4—H4	119.3
N2^i^—Zn1—N1	102.82 (12)	C3—C4—H4	119.3
C1—N1—Zn1	124.6 (3)	C1—C5—H5A	109.5
C1—N1—C4	118.7 (3)	C1—C5—H5B	109.5
C4—N1—Zn1	116.7 (3)	C1—C5—H5C	109.5
C2—N2—Zn1^ii^	124.2 (3)	H5A—C5—H5B	109.5
C2—N2—C3	119.2 (3)	H5A—C5—H5C	109.5
C3—N2—Zn1^ii^	116.5 (3)	H5B—C5—H5C	109.5
N1—C1—C2	120.3 (3)	C2—C6—H6A	109.5
N1—C1—C5	119.5 (3)	C2—C6—H6B	109.5
C2—C1—C5	120.2 (3)	C2—C6—H6C	109.5
N2—C2—C1	119.7 (3)	H6A—C6—H6B	109.5
N2—C2—C6	118.8 (3)	H6A—C6—H6C	109.5
C1—C2—C6	121.5 (4)	H6B—C6—H6C	109.5
			
Zn1—N1—C1—C2	179.3 (3)	C1—N1—C4—C3	−0.5 (6)
Zn1—N1—C1—C5	−2.2 (5)	C2—N2—C3—C4	−0.8 (6)
Zn1—N1—C4—C3	178.3 (3)	C3—N2—C2—C1	−1.7 (5)
Zn1^ii^—N2—C2—C1	175.4 (3)	C3—N2—C2—C6	179.6 (4)
Zn1^ii^—N2—C2—C6	−3.4 (5)	C4—N1—C1—C2	−2.0 (6)
Zn1^ii^—N2—C3—C4	−178.1 (3)	C4—N1—C1—C5	176.5 (4)
N1—C1—C2—N2	3.2 (6)	C5—C1—C2—N2	−175.3 (4)
N1—C1—C2—C6	−178.2 (4)	C5—C1—C2—C6	3.4 (6)
N2—C3—C4—N1	2.0 (6)		

Symmetry codes: (i) −*x*+*y*+1, −*x*+1, *z*+1/3; (ii) −*y*+1, *x*−*y*, *z*−1/3.

### catena-Poly[[dichloridozinc(II)]-µ-2,3-dimethylpyrazine-κ2N1:N4] (2) Hydrogen-bond geometry (Å, º)

**Table:**

*D*—H···*A*	*D*—H	H···*A*	*D*···*A*	*D*—H···*A*
C3—H3···Cl1^iii^	0.94	2.90	3.590 (4)	132
C3—H3···Cl2^ii^	0.94	2.85	3.482 (4)	126
C4—H4···Cl1	0.94	2.87	3.500 (4)	126
C5—H5*A*···Cl2	0.97	2.85	3.724 (5)	151
C6—H6*A*···Cl1^ii^	0.97	2.80	3.721 (5)	160
C6—H6*C*···Cl2^iv^	0.97	2.80	3.596 (5)	140

Symmetry codes: (ii) −*y*+1, *x*−*y*, *z*−1/3; (iii) −*y*, *x*−*y*−1, *z*−1/3; (iv) −*y*+1, *x*−*y*+1, *z*−1/3.
